# Complexity of Body Movements during Sleep in Children with Autism Spectrum Disorder

**DOI:** 10.3390/e23040418

**Published:** 2021-03-31

**Authors:** Naoki Furutani, Tetsuya Takahashi, Nobushige Naito, Takafumi Maruishi, Yuko Yoshimura, Chiaki Hasegawa, Tetsu Hirosawa, Mitsuru Kikuchi

**Affiliations:** 1 Department of Psychiatry and Neurobiology, Graduate School of Medical Science, Kanazawa University, Kanazawa 920-8640, Japan; nobunobu@med.kanazawa-u.ac.jp (N.N.); hirosawatetsu1982@yahoo.co.jp (T.H.); mitsuruk@med.kanazawa-u.ac.jp (M.K.); 2 Research Center for Child Mental Development, Kanazawa University, Kanazawa 920-8640, Japan; yukuchen@staff.kanazawa-u.ac.jp (Y.Y.); hasegawachiaki1014@gmail.com (C.H.); 3 Department of Neuropsychiatry, University of Fukui, Fukui 910-1193, Japan; 4 Uozu Shinkei Sanatorium, Uozu 937-0017, Japan; 5 Department of Earth and Planetary Sciences, Graduate School of Sciences, Kyushu University, Fukuoka 819-0395, Japan; maruishi.takafumi.576@s.kyushu-u.ac.jp; 6 Institute of Human and Social Sciences, Kanazawa University, Kanazawa 921-1192, Japan

**Keywords:** actigraphy, accelerometer, circadian rhythm disruption, detrended fluctuation analysis (DFA), insomnia, entropy-based methods, expanded sample entropy (expSampEn), false nearest neighbors (FNN), information theory, long-range temporal correlation

## Abstract

Recently, measuring the complexity of body movements during sleep has been proven as an objective biomarker of various psychiatric disorders. Although sleep problems are common in children with autism spectrum disorder (ASD) and might exacerbate ASD symptoms, their objectivity as a biomarker remains to be established. Therefore, details of body movement complexity during sleep as estimated by actigraphy were investigated in typically developing (TD) children and in children with ASD. Several complexity analyses were applied to raw and thresholded data of actigraphy from 17 TD children and 17 children with ASD. Determinism, irregularity and unpredictability, and long-range temporal correlation were examined respectively using the false nearest neighbor (FNN) algorithm, information-theoretic analyses, and detrended fluctuation analysis (DFA). Although the FNN algorithm did not reveal determinism in body movements, surrogate analyses identified the influence of nonlinear processes on the irregularity and long-range temporal correlation of body movements. Additionally, the irregularity and unpredictability of body movements measured by expanded sample entropy were significantly lower in ASD than in TD children up to two hours after sleep onset and at approximately six hours after sleep onset. This difference was found especially for the high-irregularity period. Through this study, we characterized details of the complexity of body movements during sleep and demonstrated the group difference of body movement complexity across TD children and children with ASD. Complexity analyses of body movements during sleep have provided valuable insights into sleep profiles. Body movement complexity might be useful as a biomarker for ASD.

## 1. Introduction

Autism spectrum disorder (ASD) is a neurodevelopmental disorder characterized by difficulties with social interaction and communication [1]. Many results of earlier studies have demonstrated that children with ASD commonly experience sleep problems [2,3,4], and that they are more likely to persist than in typically developing (TD) children [5,6,7,8,9]. Sleep disturbances reportedly correlate with core ASD symptoms [4,10,11,12,13,14,15,16] and other co-occurring symptoms and behaviors [3,10,15,16,17,18], such as anxiety [13,14,19], withdrawal [20,21], attention, hyperactivity, and aggression [12,14,15,22,23].

Polysomnography (PSG), regarded as the gold standard for sleep quality assessment, records many physiological parameters, including electroencephalography, electrooculography, electromyography, and electrocardiography, respiratory measures, and leg muscle activity. However, many ASD children with sensory abnormalities might not tolerate multiple electrodes on their body during sleep [24,25,26]. As an alternative, actigraphy, which measures body movement acceleration, has been used as a non-invasive and objective tool to assess the sleep–wake cycle. Recently, good agreement between actigraphy and PSG has been reported from sleep assessments of children with ASD [25]. Earlier studies of children with ASD have mainly found longer sleep latency, circadian rhythm disruption, lower sleep efficiency, and shorter total sleep time [2,3,4,17,27]. However, across individuals, children with ASD experience different sleep difficulties such as insomnia, circadian rhythm disorder, and parasomnia. Such difficulties might be attributable to various factors such as circadian-relevant gene anomalies, abnormal melatonin rhythms, brain wave organization, hyper-arousal, sensory hyper-reactivity, and various stresses, suggesting great heterogeneity of sleep problems in children with ASD [3,16]. Given that context, assessing the complex dynamics of body movements is expected to be useful in addition to assessing simple sleep parameters. In fact, the importance of nonlinear analyses and feature extraction have been reported from an actigraphy study [28]. Moreover, some earlier studies have shown significant alterations of movement complexity in patients with bipolar disorder [29,30,31,32,33], dementia [34,35], and insomnia [36].

Physiological systems are complex dynamical systems that integrate various mechanisms with various spatial and temporal scales. To date, the complexities of various physiological data such as EEG, ECG, respiration, pulse, and DNA sequences have been investigated. An exceedingly common complexity analysis of a one-dimensional time series is a deterministic approach that evaluates chaotic attractors. According to Takens’ embedding theorem, when a sequence consists of scalar measurement of a deterministic dynamical system [37], the time delay coordinate map with a sufficiently large dimension of the sequence provides a one-to-one image of the overall system behavior. Chaotic attractors are evaluated using two approaches with the delay coordinate: geometrically by fractal dimensions of the attractors and dynamically by divergence of nearby trajectories [38]. The latter one characterizes the sensitivity to initial conditions, which is an important aspect of complex systems. Another approach to characterize this sensitivity is the use of information-theoretic measures [39]. This type of nonlinear analysis, such as approximate entropy (ApEn) and sample entropy (SampEn), is a stochastic approach to quantify the diffusion of nearby trajectories. In other words, these measures evaluate signal variation by quantifying the irregularity and unpredictability of the time series. It is important to measure the entropy on multiple time scales to consider the hierarchical structures of physiological bases [40,41]. Compared to investigation of strange attractors, the information-theoretic measures are better suited for analyzing short, noisy, and non-stationary sequences, which are common in physiological data [42]. Physiological processes are non-stationary. Therefore, it is also meaningful to examine temporal changes in complexity. Our earlier work showed that our proposed expanded sample entropy (expSampEn), which quantifies the unpredictability of each time point, improved neural decoding accuracy [43]. In addition, other nonlinear analyses have specifically examined longer-range temporal relations. Fluctuation analysis quantifies the fractality of a cumulative summed sequence to ascertain the long-range temporal correlation in the original time series [44,45].

For this study, we applied several complexity analyses to data of body movements during sleep. This report is the first of a study characterizing the complexity of body movement in TD children and children with ASD. In summary, our aims were two-fold: (1) describe details of the complex dynamics of body movements, and (2) demonstrate group differences of complexity of body movements across TD children and children with ASD. To this end, we first examined the presence of determinism using the false nearest neighbor (FNN) method. Then, we evaluated sensitivity to initial conditions using ApEn, SampEn, and expSampEn, and evaluated temporal correlation using detrended fluctuation analysis (DFA).

## 2. Materials and Methods

### 2.1. Participants, Measurements, and Data Preprocessing

Information related to participants, measurements, and preprocessing of actigraphy has been reported by Naito et al. [46]. Participants in this cross-sectional study were 17 TD children with no reported behavioral or language problem and 17 children with ASD recruited from Kanazawa University and prefectural hospitals in Kanazawa or Toyama. Children in the TD and ASD groups were excluded from the study if a review of their medical history showed a history of epilepsy or intellectual disability or if they were taking psychotropic medications. Children with ASD were diagnosed using the Diagnostic and Statistical Manual of Mental Disorders (5th edition) (DSM-5) [1], the Japanese translation of the Diagnostic Interview for Social and Communication Disorders (DISCO) [47,48], or the Japanese translation of the Autism Diagnostic Observation Schedule (ADOS) [49,50,51]. All children fulfilled the diagnosis of childhood autism or atypical autism with DISCO and autism spectrum disorder with DSM-5. All except one also met the ADOS criteria for autism or autism spectrum disorder (Table 1). Children with psychiatric disorders other than ASD and ADHD were excluded using the Mini International Neuropsychiatric Interview for Children and Adolescents [52]. As presented in Table 2, no significant difference was found between TD and ASD groups in terms of gender, age, usual sleep duration, or sleep quality (rating scale: 1 = very bad, 2 = bad, 3 = somewhat bad, 4 = somewhat good, 5 = good, and 6 = very good) as reported by caregivers, or in cognitive skills as assessed by the Japanese adaptation of the Kaufman Assessment Battery for Children (K-ABC) [53,54]. Yuko Yoshimura, a speech language hearing therapist, and Chiaki Hasegawa, a psychologist, administered the psychological tests. The Ethics Committee of Kanazawa University Hospital approved the methods and procedures, which were performed in accordance with the Declaration of Helsinki. Parents agreed to their child’s participation in the study with full knowledge of the experimental characteristics of the research. Written informed consent was obtained after a complete explanation of this study, but before participation in the study.

Body movement was measured using a wristwatch-like accelerometer (Gen-2 GSR Wristband; Interuniversity Microelectronics Centre, Leuven, Kingdom of Belgium) attached to the waist [46]. The acceleration was measured and sampled at 32 Hz from immediately before the child entered the bed to immediately after the child exited the bed. Body movements were measured on at least three nights, excluding days when the child had a sickness such as a common cold.

The acceleration time series were preprocessed using software (Brain Vision Analyzer; Brain Products GmbH, Gilching, Germany) and Python. The three-dimensional (3D) time series recorded at a sampling rate of 32 Hz was resampled to 1 Hz by block averaging over 1 s, high-pass filtering at 0.0028 Hz in each dimension to exclude the effect of sustained gravitational acceleration, and conversion to a one-dimensional (1D) time series by calculating the root mean square (RMS) for each time point. We analyzed data of 8 h after the first time the acceleration became less than 0.1 G/s for 10 min.

### 2.2. Surrogate Analysis

**Figure 1:**
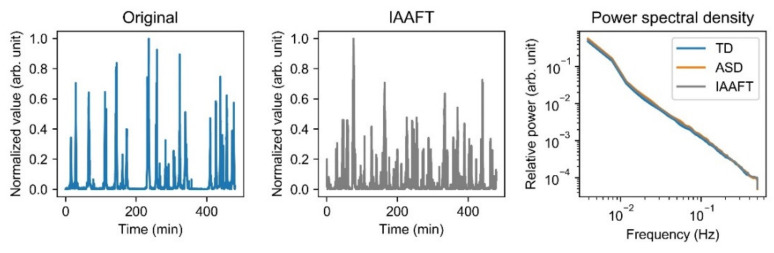
Surrogate data generated by the iterative amplitude-adjusted Fourier transform (IAAFT) algorithm: examples of original time series (**left**) and its surrogate (**middle**). The mean power spectral density (PSD) of TD, ASD, and surrogates (**right**). No significant difference of PSD was found among TD (blue), ASD (orange), and surrogates (gray). TD, typically developing children; ASD, children with autism spectrum disorder.

To confirm the body movement nonlinearity, we also performed surrogate analysis using the iterative amplitude-adjusted Fourier transform (IAAFT) algorithm [55,56]. This algorithm is proposed for testing of the null hypothesis that the time series has been generated by a stationary linear stochastic process with Gaussian inputs that has possibly gone through a static monotonic transformation during measurement. This surrogate series has the same linear correlation and probability distribution as the original time series. Rejection of the null hypothesis indicates the existence of nonlinearity in the time series [38]. We generated 20 surrogates for each time series and examined their power spectral density (PSD) using Welch’s method (Figure 1). No significant difference was found for PSD among TD, ASD, and the surrogates.

### 2.3. Raw and Thresholded Data

**Figure 2:**
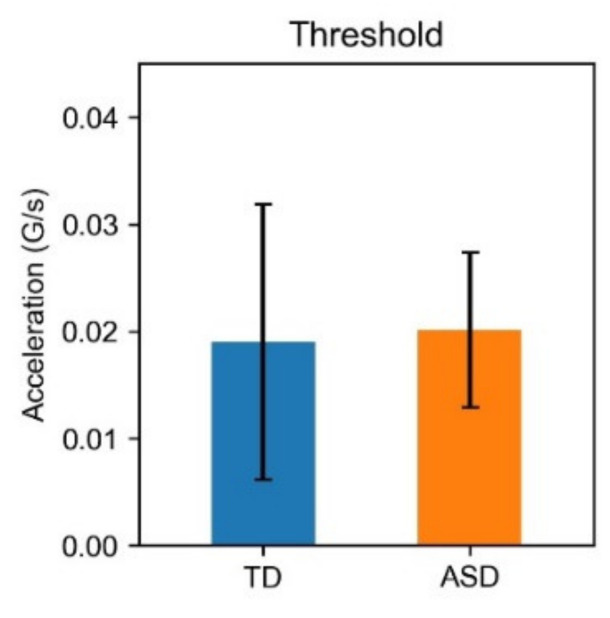
Thresholds of TD children and children with ASD. The median value of each time series was used as the threshold. No significant difference was found between TD and ASD. Error bars represent the standard deviation of the thresholds.

Additionally, time series of two types were applied to the complexity analyses. In conventional actigraphs, the activity is usually recorded in each epoch by algorithms of three types: summing all acceleration values (digital integration, DI), computing the number of values above a threshold (time above threshold, TAT), or computing the number of zero crossings (ZC). However, raw data can also be used [57]. The DI and raw data include information about the magnitude of acceleration. For TAT and ZC, the information is converted to the iterations and durations of the activity, not the magnitude of acceleration. Therefore, we also used raw and thresholded data to analyze information of two types: magnitude of activity and appearance of activity. To exclude the influence of inter-subject and inter-day variation in the magnitude of body movements, we binarized body movements using the median value for each subject and each night. No significant difference of the threshold was found between TD children and children with ASD (Figure 2).

### 2.4. Complexity Analyses

#### 2.4.1. Determinism Detection

**Figure 3:**
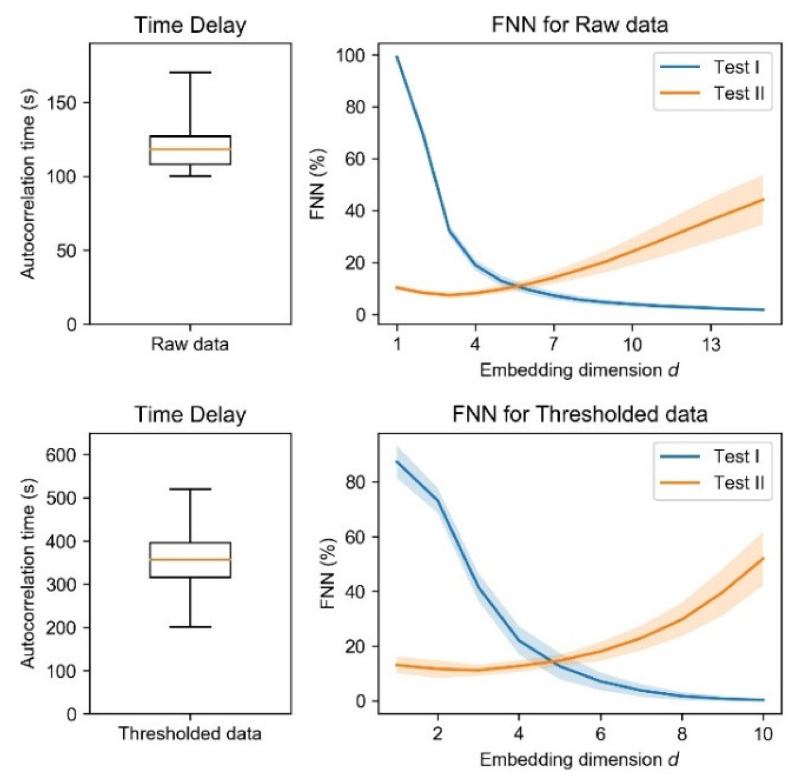
Time delay and false nearest neighbor (FNN). Upper and lower panels respectively show those of raw data and thresholded data. Left panels—Median values of autocorrelation time were set to the time delay for the FNN method (raw data, 116 s; thresholded data, 347 s). Orange lines, boxes, and whiskers respectively, indicate the median, percentiles 25 and 75, and minimum and maximum of autocorrelation time of all subjects. Right panels—Tests I and II respectively signify FNN for Equations (2) and (3) (see Section 2.4.1). The shaded areas represent the standard deviation of the fraction of FNN. For neither raw nor thresholded data did the fraction of FNN converge to zero.

To detect the determinism of body movement dynamics, we used the FNN algorithm with an appropriate time delay [38,58]. One can consider a time series = {*x_n_*, *n* = 1, …, *N*}, for which *n* represents each time point from 1 to *N* time points. As the first step in determinism detection, we computed 1/*e* decay time of the autocorrelation function (autocorrelation time), which is a common choice of appropriate time delay [38]. We used the median value of autocorrelation time as the time delay for the FNN method (Figure 3). Then, we consider *d*-dimensional delay vectors as:

(1) $$y_{i}^{\left(d\right)}\;=\;\left(x_{i},\;x_{i+\tau },\;x_{i+2\tau },\;\ldots ,\;x_{i+\left(d-1\right)\tau }\right)$$

where *d* represents the embedding dimension and τ stands for the time delay. We computed the fraction of FNN for each *d* that satisfies the following two conditions:

(2) $$\frac{\left|x_{i+d\tau }-x_{n\left(i,d\right)+d\tau }\right|}{\left\|y_{i}^{\left(d\right)}-y_{n\left(i,d\right)}^{\left(d\right)}\right\|}\;>A\;$$

(3) $$\frac{\left\|y_{i}^{\left(d+1\right)}-y_{n\left(i,d\right)}^{\left(d+1\right)}\right\|}{\sigma }\;>B$$

Therein, *n*(*i,d*) stands for the time point of the nearest neighbor of *y_i_*^(*d*)^, σ signifies the standard deviation (SD) of the scalar time series {*x_i_*}, ||∙|| represents the Euclidean norm, and A and B denote suitable thresholds. The fraction of *i* that satisfies (2) or (3) among all nearest neighbor combinations was defined as the fraction of FNN. If the time series is generated using a deterministic process, then it is expected that the chaotic attractor can be unfolded in the dimension where the fraction of FNN converges to zero. However, for a stochastic time series, it is expected that the fraction of FNN will never become zero. Herein, A and B were set as 10.0 and 2.0 [58]. The Theiler window was set to the same value as the delay, τ [38]. In addition, the inputs were moving-averaged over delay, τ, before counting the FNNs. The analyses in this section were performed using NoLiTSA, a Python module (https://github.com/manu-mannattil/nolitsa) assessed on 12 March 2021.

#### 2.4.2. Information-Theoretic Analyses

To characterize the irregularity and unpredictability of body movement dynamics, we conducted entropy-based analyses: ApEn [59], SampEn [42], and expSampEn [43]. The following is a brief summary of these algorithms [43,60]. Consider a time series $=\left\{x_{n},\;n=1,\ldots ,N\right\},$ in which *n* represents each time point and *N* denotes the total length. Defining $x_{n}^{-}=\left[x_{n-m}\ldots x_{n-1}\right]$ as the *m*-dimensional past vector, then $x_{n}=\left[x_{n-m}\ldots x_{n}\right]$ is the (*m +* 1)-dimensional vector obtained by concatenating current value *x_n_* to $x_{n}^{-}$. Also, $p\left(x_{n}|x_{n}^{-}\right)$ is the conditional probability that the current value is close to $x_{n}$ when the *m*-dimensional past vector is close to $x_{n}^{-}$. Also, $p\left(x_{n}\right)$ and $p\left(x_{n}^{-}\right)$ are the joint probabilities that *m* + 1 and *m* consecutive points in the time series are respectively close to $x_{n}$ and $x_{n}^{-}$. Generally, points or vectors within *r* × SD of Chebyshev distance are regarded as “close”. Then, ApEn, SampEn, and expSampEn are represented as:

$$\mathrm{ApEn}=-\langle \log p\left(x_{n}|x_{n}^{-}\right)〉=-\langle \log\frac{p\left(x_{n}\right)}{p\left(x_{n}^{-}\right)}〉$$

$$\mathrm{SampEn}=-\log\frac{\langle p\left(x_{n},〉\right)}{\langle p\left(x_{n}^{-}\right)〉}$$

$$\mathrm{expSampEn}\;\left(n\right)\;=-\log p\left(x_{n}|x_{n}^{-}\right)$$

where <∙> represents the average over time. Here, ApEn and SampEn are single values because they represent the irregularity of the entire time series, whereas expSampEn is a time series because it represents the irregularity of each time point with respect to the entire time series. In other words, the idea of expSampEn is based on the local or pointwise use of information theory [61,62]. In fact, the average of expSampEn over the entire time is equal to ApEn. As in our earlier work [43,63], we used *m* = 2 and *r* = 0.2 to calculate the entropy.

Additionally, the complex dynamics are expected to be described in greater detail by examining the overall picture of these information-theoretic measures over multiple time scales [40,41]. Therefore, we calculated ApEn, SampEn, and expSampEn for three time scales (30, 100, and 300 s). The inputs were moving-averaged over the time scales before calculating ApEn, SampEn, and expSampEn. The threshold of nearest neighbor, *r* × SD, was defined for the moving-averaged time series of each time scale. The analyses explained in this section were performed using EntroPy, a Python module (https://github.com/raphaelvallat/entropy) assessed on 12 March 2021.

#### 2.4.3. Fluctuation Analysis

To examine the long-range temporal correlation of body movement dynamics, we performed fluctuation analysis that is able to quantify the fractality of a cumulative summed sequence. We applied DFA (detrended fluctuation analysis): a very common fluctuation analyses [44,45]. In this algorithm, the entire series is first split by size, *n*. Then, the root mean square of the deviation from the local trend is computed to show typical fluctuations of the series, *F*(*n*):

$$F\left(n\right)=\sqrt{\frac{1}{N}\overset{N}{\underset{k=1}{\sum }}{\left(y\left(k\right)-y_{n}\left(k\right)\right)}^{2}}$$

In that equation, *y* is the cumulative summed sequence of *x*, and *y_n_*(*k*) is the local trend. *F*(*n*) usually exhibits a power law scaling $F\left(n\right)\;\propto n^{\alpha }$. Temporal correlation is evaluated by its exponent α. When α ≈ 1/2, *x* is temporally uncorrelated, and when α > 1/2, *x* is temporally correlated. When α ≈ 1, *F*(*n*) exhibits 1/*f* scaling, and *x* is temporally long-range correlated [36]. We examined the scaling properties of *y* between 5 min and 2 h.

### 2.5. Statistical Analyses

Student’s *t*-tests were applied to all two-group comparisons. ExpSampEn is a time series. Therefore, we did not correct for multiple comparisons in expSampEn. To ensure the same conditions as those used for expSampEn, we also did not correct for multiple comparisons in any other two-group comparison. Additionally, because of the large variation of expSampEn, a moving average for the 60 min preceding the *t*-tests was taken.

Spearman’s correlation coefficients were calculated to examine the correlation between the original time series (raw data and thresholded data) and expSampEn (Table 3). All time series were moving-averaged for the 60 min before calculating the correlation coefficients.

For results of all statistical analyses, *p* < 0.05 was inferred as indicating statistical significance.

## 3. Results

### 3.1. Stationarity of Body Movements Overnight

Initially, to test the stationarity of the body movement time series, we compared the distribution of the first half of the time series with the distribution of the entire time series [38,64]. We conducted a chi-square test using five bins for the raw data and two bins for the thresholded data. Results showed that all 34 subjects × 3 times of the raw data and 92.2% (except eight data) of the thresholded data were rejected for stationarity at the 0.05 level of significance. These results indicate that the distributions of both the magnitude and the presence of body movements are significantly different between the first and latter half of sleep, i.e., the magnitude and the presence of body movements were non-stationary overnight.

### 3.2. Deterministic Chaos

Next, to detect the deterministic chaos of body movement dynamics, we used the FNN algorithm. According to Takens’ embedding theorem, detecting determinism requires an appropriate time delay [38]. We applied median values of the autocorrelation time as the time delay (raw data, 116 s; thresholded data, 347 s; Figure 3, left panels). As shown in the right panels of Figure 3, the fraction of FNN did not converge to zero for either the raw and thresholded data, indicating that deterministic chaos was not detected using the FNN algorithm. However, this lack of convergence does not signify that the body movement is not a complex system. Rather, the chaos cannot be detected because the deterministic approach is vulnerable to noise and non-stationarity. Actually, stationarity was rejected for much of the time series, as described in Section 3.1. Therefore, we investigated body movement complexity using information theoretic measures and fluctuation analysis, which are robust to noise and non-stationarity, as described in the following sections.

### 3.3. Information-Theoretic Measures

**Figure 4:**
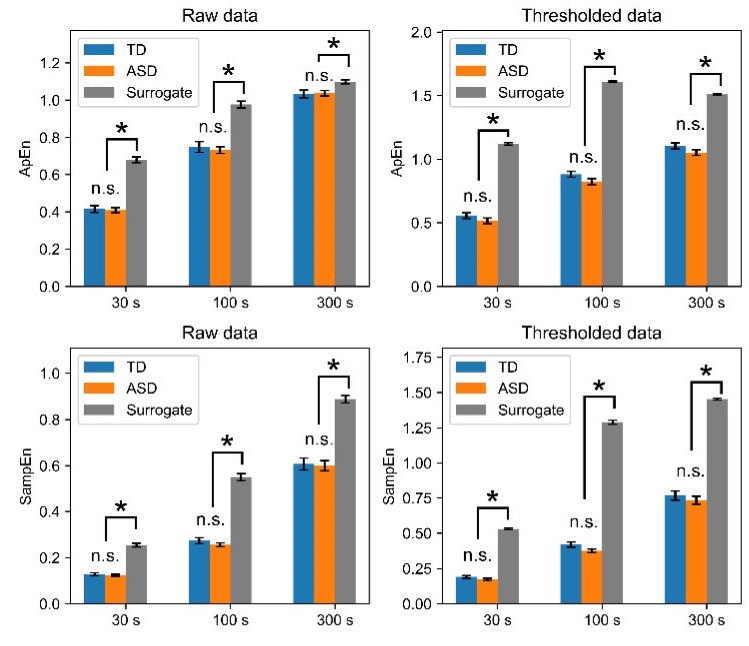
ApEn and SampEn for raw and thresholded data. Time scales are 30, 100, and 300 s. No significant difference between TD children and children with ASD was found for either raw or thresholded data on any time scale, although significant differences were found between original and surrogate data. ApEn, approximate entropy; SampEn, sample entropy; n.s., not significant (*p* ≥ 0.05); *, *p* < 0.001.

We then applied information theoretic measures to quantify the irregularity and unpredictability associated with sensitivity to initial conditions [39]. First, we examined the irregularity of the time series of data for the entire night using ApEn and SampEn. No significant difference was found in ApEn or SampEn between TD children and children with ASD for either raw or thresholded data. However, on almost all scales, ApEn and SampEn were significantly lower in the original data than in the surrogates, for which linearity was assumed (Figure 4). These results demonstrated nonlinearity in the irregularity of body movements, but the irregularity of overnight body movements was comparable between TD children and children with ASD.

**Figure 5:**
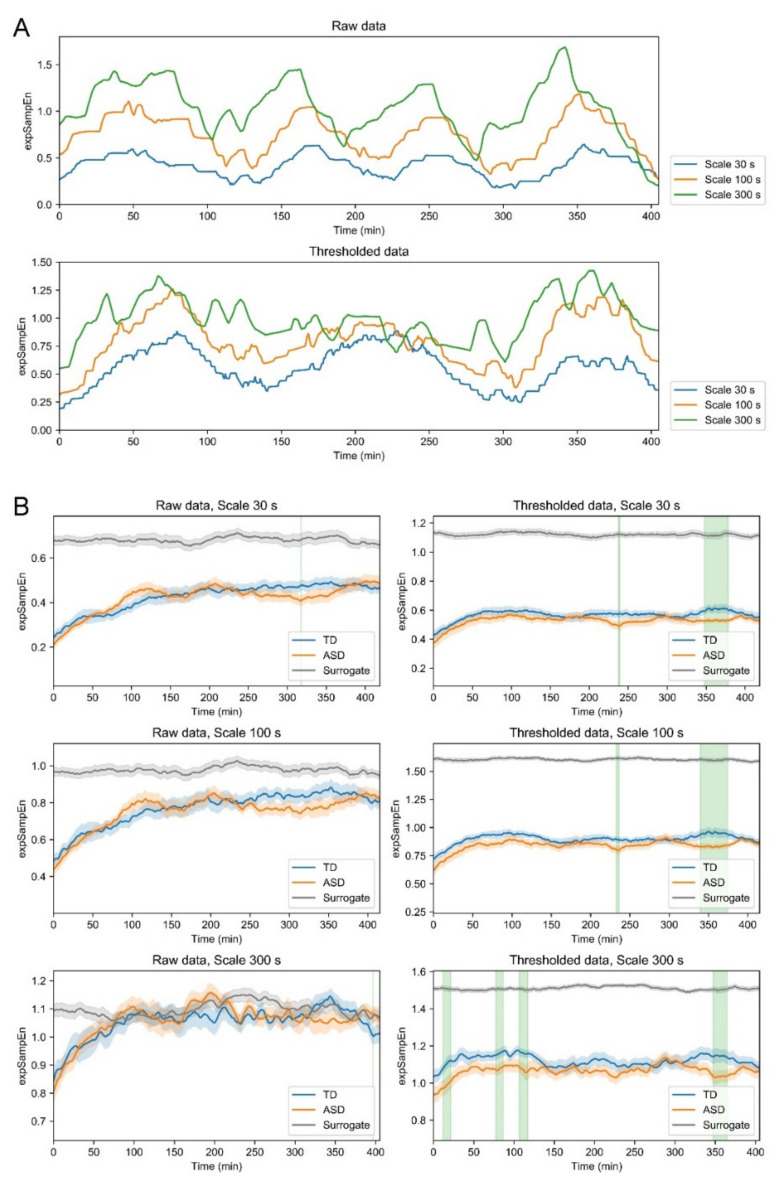
expSampEn time series are shown (moving-averaged over 60 min). (**A**) Typical examples of the expSampEn time series of raw and thresholded data. Both are measured on the same day for the same subject. (**B**) Left and right panels respectively show expSampEn for raw data and thresholded data. Time scales are 30 (upper panels), 100 (middle panels), and 300 s (lower panels). The solid line and the shaded area respectively show the mean and the standard error. Green shading represents that the expSampEn is significantly lower in ASD than in TD (*p* < 0.05).

Next, we examined the irregularity and unpredictability at each time point using expSampEn, which is the local version of ApEn and SampEn [43,61]. We initially checked for temporal changes in irregularity of each subject of each night. Although both the expSampEn of raw data and of thresholded data showed phasic behaviors, the behaviors are apparently uncorrelated (Figure 5A). As described in Table 3, although the raw data and thresholded data were highly correlated (*R* = 0.88–0.89), expSampEn of the thresholded data showed weaker correlation with raw data (*R* = 0.14–0.25) and with expSampEn of raw data (*R* = 0.33–0.51). This result is apparently mainly attributable to the weak correlation between the thresholded data and their expSampEn (*R* = 0.23–0.46). Subsequently, we investigated group differences in expSampEn between TD and ASD (Figure 5B). Although almost no significant difference was found in the raw data, expSampEn in the thresholded data yielded significantly lower results for ASD than for TD for 300 s from sleep onset to 2 h after sleep onset, and for 30, 100, and 300 s at approximately 6 h after sleep onset. Additionally, for several time scales, expSampEn yielded significantly lower results for the original data than for the surrogates.

To evaluate group differences of irregularity, we compared the representative values of TD and ASD obtained for high- and low-irregularity states. The 90th and 10th percentiles of the moving-averaged expSampEn for thresholded data were defined respectively as the representative values for the high-irregularity and low-irregularity states because the degree of irregularity behaves phasically (Figure 5A), and because the timings of switching between the high-irregularity and low-irregularity states differ among subjects. The high-irregularity values of ASD on the time scales of 100 and 300 s were significantly lower than those of TD, although no significant difference was found for the low-irregularity value (Table 4).

In summary, children with ASD moved less irregularly than TD children in the early (up to 2 h) and late (after 5 h) periods of sleep. From a periodical view of irregularity, differences in irregularity between TD and ASD were observed at the high-irregularity state. Additionally, nonlinearity was involved in the irregularity of body movements.

### 3.4. DFA

**Figure 6:**
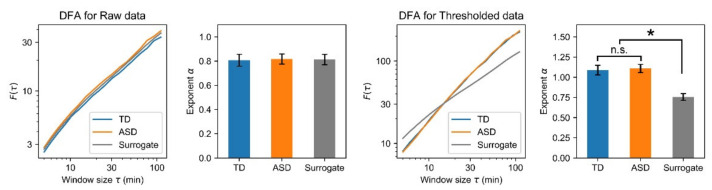
DFA for raw and thresholded data. The log–log plot shows the mean of *F*(τ). The bar plot shows the mean ± SD of exponent α. Exponent α of surrogates were significantly lower than that of TD and ASD children for thresholded data. n.s., not significant (*p* ≥ 0.05); * *p* < 0.001.

To characterize the temporal correlation of body movement dynamics, we applied DFA to the cumulative summed sequence of raw and thresholded data (Figure 6). We confirmed power-law scaling for at least one order of time duration and evaluated exponent α by fitting it. No significant difference was found between exponent α of TD children and children with ASD, although exponent α of surrogates was significantly lower than that of TD children and children with ASD for thresholded data (*p* < 0.001). Exponent α of the original data (especially the thresholded data) was approximately 1.0, suggesting that fluctuation of the original data exhibits 1/*f* scaling. In other words, body movements during sleep are not temporally discrete but are rather temporally correlated. This temporal correlation was similar between TD children and children with ASD.

## 4. Discussion

Earlier reports of some studies have described high prevalence of sleep difficulties of various types across children with ASD [2,3] and have suggested the involvement of various neurophysiological mechanisms [3,16]. To characterize this diverse ASD-related sleep difficulty, assessment of body movement complexity during sleep might add another dimension to sleep difficulties already identified from conventional sleep assessments, such as sleep latency and total sleep time [24]. Many physiological phenomena can be described in terms of complex dynamics. Body movements are also expected to have complexity. In fact, complexity analyses have been applied to some earlier actigraphy studies [29,30,31,32,33,34,35,36,65,66]. Nevertheless, earlier studies have yielded no consistent findings across various analyses of complexity. This report is the first of a detailed study comprehensively investigating body movement complexity during sleep using different complexity measures and highlighting their relevance to ASD diagnosis. To examine the validity of complexity analyses, we first discuss the complex dynamics of body movements during sleep, irrespective of the psychiatric disorder. Subsequently, we characterized the ASD-related complexity profiles of body movements. Finally, the study limitations are listed.

### 4.1. Complex Dynamics in Body Movements

Initially, body movement complexity during sleep, irrespective of psychiatric disorders, is discussed. As explained earlier in Section 2.3, raw and thresholded data were used respectively, to examine the complexity profile of the magnitude and presence of body movement. Additionally, body movement nonlinearity was investigated using surrogates. In summary, although we were unable to detect determinism (Figure 3), results showed that nonlinear processes reduced the irregularity and unpredictability (Figure 4 and Figure 5B) and enhanced long-range temporal correlation (Figure 6) in both the magnitude and presence of body movement. Strong non-stationarity, as described in Section 3.1, and the influence of nonlinear processes were observed in body movements during sleep, suggesting that information-theoretic analyses and fluctuation analyses, which are robust to non-stationary data, are effective for investigating complex systems of body movement.

Regarding temporal correlation, earlier studies applying DFA analysis to actigraphy have also revealed the existence of 1/*f* fluctuations in body movements during daytime [67], night [36], and 24 h [29,34,35]. These findings suggest that body movement during sleep shows long-range temporal correlation and suggest that it can be modeled by self-similar stochastic processes such as fractional Brownian motion [68]. Additionally, although entropy-based measures cannot be compared directly because of their relativity [69], altered irregularity in body movement has been reported for various psychiatric disorders, as described specifically hereinafter. These observations also suggest that body movement complexity can be characterized by irregularity. Surrogate analyses in both information-theoretic measures and DFA suggest the influence of nonlinear processes in body movement. The only report describing a study that compares body movement time series with their surrogates using fluctuation analysis explains the existence of nonlinear processes in body movement fluctuations in healthy subjects [70]. Consideration of these findings together suggests that body movements can be modeled using a nonlinear process with self-similarity that is more complex than fractional Brownian motion.

### 4.2. Group Differences (TD vs. ASD) in Complexity of Body Movement

Results of group comparison of the body movement complexity demonstrated less irregularity in ASD than in TD, in a time-specific manner (Figure 5B). Earlier reports of some studies have described increased wake after sleep onset based on caregiver’s reports [8], PSG [71,72,73], and actigraphy [74], suggesting increased body movement after sleep onset in ASD. Use of the same dataset as that used for the present study revealed that the duration of body movements was longer in children with ASD over similar time periods to those examined for our study [46]. Considering these findings collectively, the behavior of increased body movement in ASD is characterized with regularity, specifically in the early and late stages of sleep. It is noteworthy that, despite the high correlation across original datasets (raw vs. thresholded data), complexity analysis applied to them indicated different and distinct characteristics (Table 3). This finding might be the reason for the low complexity of thresholded data in spite of the increased body movement in children with ASD. Additionally, when we defined high-irregularity and low-irregularity states to incorporate consideration of the phasic behavior of irregularity (Figure 5A) and compared the respective states for TD and ASD, reduced irregularity of body movement in ASD was found only in the high-irregularity state. This result suggests the importance of phase (e.g., sleep stage or gene expression) for elucidating the sleep characteristics of ASD.

Few reports of the relevant literature have described studies examining the complexity of body movements in psychiatric disorders. For example, irregularities assessed by entropy-based methods such as SampEn are reportedly higher for mania than for bipolar depression [30,33]. Another study of schizophrenia showed a disease-associated increase of body movement [65]. However, most actigraphy studies using these information-theoretic analyses have specifically examined the active periods. No report of the relevant literature has specifically examined the sleep period. Additionally, no report of the relevant literature has described an examination of the irregularity and unpredictability of body movements in children with ASD. Compared to entropy analyses, the DFA algorithm requires long time series data aimed at evaluating long-range temporal correlation. In the only reported study analyzing fluctuations during sleep, exponent α was found to increase, showing long-range temporal correlation for insomnia patients [36]. Other reports of the literature have described that exponent α decreases with age and in Alzheimer’s disease patients [34,67], and that scale-dependent changes in exponent α are found in cases of bipolar disorder [29]. Earlier actigraphy reports have described altered irregularity and unpredictability during active periods in bipolar disorder and schizophrenia, along with altered temporal correlation in insomnia, aging, Alzheimer’s disease, and bipolar disorder. Novel findings of the present study are that the appearance of body movements during sleep in ASD is less irregular than in TD, suggesting the usefulness of entropy-based analyses of body movement during sleep for the diagnosis of ASD. However, it seems contradictory that no disruption was found in the fractality of body movement during sleep in children with ASD, who experience many sleep difficulties, despite altered fractality in the fluctuation of body movements in insomnia patients. This finding might be explained by the heterogeneity of sleep problems in children with ASD [3,16], or by differences in the ages of subjects (18–65 years in the study of Holloway et al. [36], 5–8 years in this study). Therefore, additional studies must be conducted to assess the relevance of ASD itself and sleep problems in children with ASD to the complexity of body movements.

### 4.3. Limitations

Body movement complexity exhibited phasic behavior (Figure 5A) that resembles a periodic pattern in the sleep architecture. To date, no consistent conclusion has been reported for the sleep architecture of children with ASD [75,76]. For instance, reports of earlier studies have described both increased and decreased rapid eye movement (REM) sleep [20,26,71,77,78] and slow wave sleep (SWS) [20,26,73,78]. Moreover, alteration of the cyclic alternation pattern (CAP) [27] and increased undifferentiated sleep [26,77] have been reported. For the present study, we detected a difference in the certain state of the periodic pattern during sleep between children with ASD and TD children (Table 4). It is particularly interesting, however, that two aspects of complexity (magnitude of body movement (raw data) and presence of body movement (thresholded data)) oscillated differently (Table 3). This result suggests that body movement has at least two oscillatory components. Although accelerometry alone is not regarded as able to classify sleep stages because it is an indirect measure of sleep [79,80], a recent report has described that actigraphy combined with cardiorespiratory cues can classify wake/REM/non-REM with high accuracy [81]. However, because we did not measure PSG, the relation between REM/non-REM cycle and the two types of periodic behavior of irregularity we detected remains unknown. Additional studies using both actigraphy and PSG must be conducted to identify the meanings of the periodic behaviors of complexity.

Additionally, surrogate analyses suggest that body movement is a complex system generated by nonlinear processes rather than by linear stochastic processes. However, because the null hypothesis of IAAFT assumes a stationary linear stochastic process in a precise sense, the non-stationarity of body movements (see Section 3.1) might affect the surrogate behavior. Therefore, caution is necessary when interpreting the results of surrogate analyses.

For this study, the accelerometer was attached to the waist because the comfort of children was prioritized. Another report described that even 1.5-year-old TD children placed the accelerometer on their waists for 7 consecutive days [82]. However, some reports of earlier studies have described differences of actigraphy depending on the accelerometer attachment site [83,84,85]. Additional studies must be conducted to validate the actigraphy data obtained using waist-attached accelerometers.

## 5. Conclusions

As described herein, we were unable to prove the presence of determinism using the FNN algorithm. However, the irregularity and unpredictability evaluated using entropy-based analyses and the exponent α of fluctuation analysis showed significant differences between the original data and its surrogates, suggesting that body movements become less irregular and become temporally long-range correlated by nonlinear processes. Additionally, the irregularity of the magnitude and the presence of body movement behaved phasically, but differently from one another. Within these complexities, the irregularity and unpredictability of the presence of body movements was found to be significantly lower in children with ASD than in TD children, especially during high-irregularity periods. Further investigation using PSG will be needed to determine whether body movement complexity in children with ASD is reduced only during sleep, and if so, at which sleep stage.

## Figures and Tables

**Table 1:** Autism Diagnostic Observation Schedule (ADOS) scores of children with ASD (*N* = 17).

	Score (Mean ± SD)	ASD Cut-Off
ADOS-G, Module 1 (*N* = 2)		
Communication	4.5 ± 0.7	2
Reciprocal Social Interaction	8.0 ± 4.2	4
Communication + Social Interaction	12.5 ± 3.5	7
Play	2.5 ± 2.1	
Stereotyped Behaviors and Restricted Interests	1.5 ± 0.7	
ADOS-G, Module 2 (*N* = 6)		
Communication	4.7 ± 2.2	3
Reciprocal Social Interaction	8.8 ± 1.9	4
Communication + Social Interaction	13.5 ± 3.9	8
Imagination/Creativity	1.0 ± 0.6	
Stereotyped Behaviors and Restricted Interests	1.5 ± 0.8	
ADOS-G, Module 3 (*N* = 1)		
Communication	4	2
Reciprocal Social Interaction	9	4
Communication + Social Interaction	13	7
Imagination/Creativity	0	
Stereotyped Behaviors and Restricted Interests	1	
ADOS-2, Module 2 (*N* = 6)		
Social Affect	9.0 ± 1.4	
Restricted and Repetitive Behavior	1.5 ± 1.2	
Total score	10.5 ± 2.2	8
ADOS-2, Module 3 (*N* = 2)		
Social Affect	5.5 ± 2.1	
Restricted and Repetitive Behavior	1.0 ± 0.0	
Total score	6.5 ± 2.1	7

ADOS-G, Autism Diagnostic Observation Schedule–Generic; ADOS-2, Autism Diagnostic Observation Schedule–2.

**Table 3:** Spearman’s correlation coefficient between original data and expSampEn (mean ± SD).

Data Type–Data Type	Time Scale		
	30 s	100 s	300 s
Raw–Thr	0.88 ± 0.05	0.88 ± 0.05	0.89 ± 0.06
Raw–Raw_expSampEn	0.89 ± 0.05	0.78 ± 0.06	0.41 ± 0.13
Thr–Thr_expSampEn	0.46 ± 0.15	0.46 ± 0.16	0.23 ± 0.17
Raw–Thr_expSampEn	0.22 ± 0.21	0.25 ± 0.20	0.14 ± 0.20
Raw_expSampEn–Thr_expSampEn	0.33 ± 0.16	0.49 ± 0.14	0.51 ± 0.14

Raw, raw data; Thr, thresholded data. All time series were moving-averaged over 60 min before calculating the correlation coefficients.

**Table 4:** *t*-test results for TD and ASD by high and low representative values of expSampEn.

Time Scale	90th Percentile		10th Percentile	
	*T*	*p*	*T*	*p*
30 s	1.52	0.069	0.97	0.17
100 s	2.17	0.019	0.91	0.18
300 s	1.78	0.043	1.46	0.077

**Table 2:** Demographic characteristics.

	TD	ASD	*p*-value
Number of participants	17	17	
Gender (male/female)	11/6	13/4	n.s.
Age in months, mean (range)	71.1 (61−79)	77.1 (60−98)	n.s.
Usual sleep duration (mean ± SD h)	9.45 ± 0.54	9.51 ± 0.59	n.s.
Usual sleep quality ^1^ (mean ± SD h)	5.0 ± 0.71	5.0 ± 0.87	n.s.
K-ABC Mental Processing Scale (mean ± SD)	102.8 ± 10.5	93.9 ± 18.9	n.s.

^1^ Sleep quality was rated as 1 = very bad, 2 = bad, 3 = somewhat bad, 4 = somewhat good, 5 = good, and 6 = very good. K-ABC, Kaufman Assessment Battery for Children; n.s., not significant, TD = typically developing children, ASD = Autism spectrum disorder

## Data Availability

The data underlying this article cannot be shared publicly because of a non-disclosure agreement. The data will be shared on reasonable request to the corresponding author.

## Abbreviations

ADOS-G	Autism Diagnostic Observation Schedule–Generic
ApEn	approximate entropy
ASD	autism spectrum disorder
CAP	cyclic alternation pattern
DFA	detrended fluctuation analysis
DI	digital integration
DISCO	Diagnostic Interview for Social and Communication Disorders
DSM-5	Diagnostic and Statistical Manual of Mental Disorders (5th edition)
expSampEn	expanded sample entropy
FNN	false nearest neighbor
IAAFT	iterative amplitude-adjusted Fourier transform
K-ABC	Kaufman Assessment Battery for Children
PSD	power spectral density
PSG	Polysomnography
REM	rapid eye movement
RMS	root mean square
SampEn	sample entropy
SD	standard deviation
SWS	Slow-wave sleep
TAT	time above threshold
TD	typically developing children
ZC	number of zero crossings
